# The efficacy of diode laser and subgingival air polishing with erythritol in treatment of periodontitis (clinical and microbiological study)

**DOI:** 10.1186/s12903-024-04481-6

**Published:** 2024-07-04

**Authors:** Sara M. A. Elmeligy, Wafaa Saleh, Gasser M. Elewa, Hani Z Abu El-Ezz, Noha Mostafa Mahmoud, Samah Elmeadawy

**Affiliations:** 1 Oral Medicine, Periodontology, Diagnosis and Oral Radiology Department, Faculty of Dentistry, Mansoura University, Mansoura, Egypt; 2 Oral Medicine, Periodontology, Diagnosis and Oral Radiology, Faculty of Oral and Dental Medicine, Delta University for Science and Technology, Dakahlia, Egypt; 3 Oral Medicine, Periodontology and Oral Diagnosis, Faculty of Dentistry, Suez University, Suez, Egypt; 4 Medical Microbiology and Immunology Department, Faculty of Medicine Mansoura University, Mansoura, Egypt; 5 Medical Microbiology and Immunology Department, Faculty of Medicine, Horus University, New Damietta, Egypt

**Keywords:** Periodontitis, Diode Laser, Erythritol

## Abstract

**Background:**

There is insufficient clinical and microbiological evidence to support the use of diode laser and air-polishing with erythritol as supplements to scaling and root planning(SRP). The aim of the current study is to evaluate the clinical and microbiologic efficacy of erythritol subgingival air polishing and diode laser in treatment of periodontitis.

**Methods:**

The study encompassed twenty-four individuals seeking periodontal therapy and diagnosed with stage I and stage II periodontitis. Eight patients simply underwent SRP. Eight more patients had SRP followed by erythritol subgingival air polishing, and eight patients had SRP followed by diode laser application. At baseline and six weeks, clinical periodontal parameters were measured, including Plaque Index (PI), Gingival Index (GI), periodontal Probing Depth (PPD), and Clinical Attachment Level (CAL). The bacterial count of Aggregatibacter actinomycetemcomitans(A.A), Porphyromonas gingivalis (P.G) was evaluated at different points of time.

**Results:**

The microbiological assessment revealed significant differences in the count of A.A. between the laser and erythritol groups immediately after treatment, indicating a potential impact on microbial levels. However, the microbial levels showed fluctuations over the subsequent weeks, without statistically significant differences. Plaque indices significantly decreased post-treatment in all groups, with no significant inter-group differences. Gingival indices decreased, and the laser group showed lower values than erythritol and control groups. PPD and CAL decreased significantly across all groups, with the laser group exhibiting the lowest values.

**Conclusion:**

The supplementary use of diode laser and erythritol air polishing, alongside SRP, represents an expedited periodontal treatment modality. This approach leads to a reduction in bacteria and improvement in periodontal health.

**Trial registration:**

This clinical trial was registered on Clinical Trials.gov (Registration ID: NCT06209554) and released on 08/01/2024.

## Introduction

Periodontal diseases represent a broad spectrum of oral health conditions that affect the supporting structures of the teeth, most notably the gingiva, periodontal ligament, and alveolar bone. These diseases collectively pose a significant public health challenge, impacting millions of individuals worldwide and exerting a substantial burden on overall well-being [1, 2].

At the heart of periodontal diseases lies the complex interplay between dental plaque and the body's immune response. Dental plaque, a sticky biofilm composed of bacteria, food particles, and saliva, continually forms on tooth surfaces. When plaque accumulates and is not adequately removed through oral hygiene practices such as brushing and flossing, it can give rise to an array of issues [3].

Particularly, Porphyromonas gingivalis (P.G), a Gram-negative anaerobic bacterium, has been identified in a significant proportion (85.75%) of subgingival plaques associated with chronic periodontitis. This highlights the substantial role of specific bacterial pathogens, including P.G, in the pathogenesis of periodontal disease, emphasizing the importance of targeted interventions aimed at controlling bacterial growth and restoring periodontal health. The oral cavity, home to nearly 700 distinct bacterial species, plays a central role in the development of periodontitis. Among the numerous bacterial pathogens implicated in periodontitis, Gram-negative species such as Aggregatibacter actinomycetemcomitans (A.A), P.G, Prevotella intermedia, and Tannerella forsythia are key contributors to disease progression. These bacteria form complex biofilms within dental plaque, providing a conducive environment for their growth and proliferation [4].

P.G is a Gram-negative anaerobic bacterium that is widely recognized as a key pathogen in the development and progression of periodontal disease [5]. P.G is particularly adept at forming complex biofilms within periodontal pockets, which provides it with protection from the body's immune responses and antibiotics, making it challenging to eradicate. This bacterium secretes a variety of virulence factors, including proteases and toxins, that directly contribute to tissue destruction, bone loss, and the inflammation seen in periodontal diseases. Its interactions with other oral pathogens further complicate the situation, and often leading to the exacerbation of disease severity [6].

A.A is a gram-negative, facultative anaerobic bacillus that promotes periodontal bone loss. This organism's putative virulence factors include a potent leukotoxin, lipopolysaccharide, cell surface-associated substances, enzymes, and other virulence factors that will alter the host defenses response. By using a variety of virulence factors in periodontal disease, these bacteria can cause bone resorption. Numerous studies have found that the interplay of virulence factors and the immunological response of the host frequently causes bone resorption to advance in periodontal disease [7, 8].

Exclusive reliance on mechanical therapy; Scaling and root planing (SRP) may prove insufficient in eradicating pathogenic bacterial species, particularly those harbored within anatomically inaccessible areas [9]. Recognizing these limitations, adjunctive therapies have been explored to complement mechanical debridement and enhance bacterial eradication. These supplementary modalities encompass a diverse range of approaches, including antibiotics, antiseptics, non-chemical interventions such as laser therapy, and photodynamic therapy [10].

Laser therapy, specifically utilizing the diode laser at a wavelength of 940 nm, has emerged as a promising adjunct to traditional periodontal treatment modalities like SRP. This approach offers several advantages, including enhanced healing and bactericidal effects within treated sites. The diode laser's popularity stems from its cost-effectiveness, portability, and ease of use, making it a practical option for periodontal therapy. Moreover, its ability to selectively target diseased soft tissues and microorganisms while sparing healthy surrounding tissues makes it an attractive option. The diode laser's favorable tissue penetration capabilities further contribute to its effectiveness, allowing for thorough treatment of periodontal pockets and targeted destruction of pigmented bacteria and granulation tissue [11–14].

The recently introduced air-polishing devices for periodontal treatment are evident, with a focus on low-abrasive, resorbable powders, and subgingival tools. Recent studies indicate that these devices reduce post-operative discomfort, enhance patient acceptance, and have minimal impact on surrounding tissues. Originally designed for biofilm and stain removal, the shift to resorbable powders like glycine and erythritol addresses risks associated with abrasive powders on exposed surfaces [15, 16]. Erythritol powder, a water-soluble sweetener, has gained prominence, demonstrating outcomes in periodontal treatment comparable to traditional methods [17, 18]. Studies suggest erythritol powder may have a prolonged antimicrobial effect on subgingival biofilm, potentially reducing the numbers of P.G and A.A during periodontal treatment [19].

Insufficient evidence exists regarding the impact of combining laser or erythritol powder air polishing (EPAP) alongside conventional SRP treatment in non-treated periodontitis cases, compared to conventional treatment alone. Consequently, this study aims to examine the impact on clinical and microbiological parameters resulting from the combination of laser and EPAP with SRP.

Combining diode laser therapy and erythritol air polishing with conventional SRP presents a groundbreaking approach in periodontal treatment. This innovative method targets microbial pathogens and enhances biofilm removal, aiming to improve clinical outcomes and prevent disease recurrence. Our research contributes to personalized periodontal care, tailoring treatment to individual patient needs. Through meticulous investigation, we provide valuable insights for evidence-based practice and drive innovation in periodontal therapy.

## Materials and methods

The current study involved the participation of twenty-four patients, enrolled from the Periodontology clinic at Department of Oral Medicine and Periodontology at the Faculty of Dentistry, Mansoura University and Faculty of Oral and Dental Medicine, Delta University. The selection process adhered to specific criteria. Inclusion criteria specified the inclusion of adult patients aged over 30 years, without systemic illnesses, and diagnosed with stage I & II periodontitis according to the recent classification of periodontal diseases [20]. This clinical trial was registered on Clinical Trials.gov (Registration ID: NCT06209554) and released on 08/01/2024.

Exclusion criteria included patients with systemic diseases associated with delayed wound healing, chronic smokers, those who underwent prior periodontal therapy within the last 6 months, and individuals who used antibiotics in the previous 6 months.

### Sample size calculation

Sample size calculation was based on the efficacy of diode laser and subgingival air polishing with erythritol in treatment of periodontitis based on decrease of clinical attachment level(CAL) retrieved from previous research [21]. G power program version 3.1.9.4 was utilized to calculate sample size based on effect size = 1.39, using 2-tailed test, α error = 0.05 and power = 90.0%, the total calculated sample size was 8 in each group [22].

### Randomization

Patients were then randomly distributed into three groups using a computer-generated list, ensuring allocation concealment by involving a person not associated with the study in the randomization process. The code remained undisclosed until all data had been collected, preventing the revelation of treatment groups to the clinical examiner or statistician. The randomly assigned participants with periodontitis were classified into three main groups:

Group 1 (control group): It included eight patients diagnosed with stage I & II periodontitis who received treatment consisting solely of SRP.

Group 2 (study group): It included eight patients diagnosed with stage I & II periodontitis who underwent SRP followed by the application of erythritol air polishing.

Group 3 (study group): It included eight patients diagnosed with stage I & II periodontitis who underwent SRP followed by the application of diode laser treatment.

The methodology ensured that participants received comprehensive information about the treatment procedures, covering non-surgical methods, laser application, and airflow with erythritol. The potential risks or effects associated with these procedures, along with alternative treatment options, were thoroughly explained to the participants, adhering strictly to the ethical guidelines outlined by the Faculty of Dentistry at Mansoura University, Egypt (Ethical Committee Number: A19080622). Furthermore, participants acknowledged their understanding of the explanation and expressed their legal competence to provide written informed consent before undergoing any necessary procedures. All patients underwent Phase I therapy, including SRP using ultrasonic tips and gracey curettes, along with oral hygiene instructions. Patients were advised to refrain from using any mouthwash during the study.

In our study, the focus was specifically directed towards periodontally affected teeth when measuring both clinical and microbiological parameters. This ensured a targeted assessment of the treatment's efficacy in addressing periodontal issues.

In the laser cohort, following SRP, laser application was performed exclusively on periodontitis affected teeth using a diode laser with a 940 nm wavelength (Epic X, Biolase, USA). This laser was equipped with a 300 µm uninitiated fiber tip and operated at dose (321.43 J/cm^2^), power (1.5 W), and energy (90 J) in continuous wave mode. The laser treatment targeted the periodontal pockets, extending from the bottom of the pocket to the free gingival margin, with side-to-side movements at a rate of 2 mm/second [23]. Two irradiation periods, each lasting 30 s, separated by a 60-s relaxation period, were administered [24].

In the erythritol group, we focused on teeth with periodontitis only. After SRP, a standard air polishing unit, configured per manufacturer's instructions, and employed low-abrasive powder (Erythritol 97.5%) was used. The powder chamber, filled to the indicated maximum level, ensured consistent conditions. Operating at medium water and powder settings, the air polishing device directed the jet parallel to the root's long axis, spending 5 s per surface for subgingival plaque elimination. This meticulous approach aimed to enhance periodontal health post-supra-gingival calculus removal and professional tooth cleaning [25].

The periodontal assessment including Plaque Index (PI) [26], Gingival Index (GI) [27], Periodontal Probing Depth (PPD) [28], CAL [29]. The assessment was specifically targeted periodontally affected teeth to characterize the severity of periodontitis and assess treatment outcomes accurately.

In the periodontal assessment, PPD was meticulously measured. Specifically, a six-point measurement was employed, where the probe was gently inserted into six predetermined sites around each tooth: mesio-buccal, mid-buccal, disto-buccal, mesio-lingual/palatal, mid-lingual/palatal, and disto-lingual/palatal. The deepest pocket depth per tooth was recorded for analysis, representing the most severe periodontal involvement at each tooth site. This approach provided a comprehensive evaluation of periodontal health status by identifying the areas with the greatest degree of attachment loss and probing depth, which are indicative of more advanced stages of periodontitis.

Furthermore, to calculate the mean PPD per tooth, the recorded pocket depths from all six sites around each tooth were averaged. This provided a representative value reflecting the overall periodontal condition of the tooth, considering the depth of all pockets measured. By focusing on the deepest pocket per tooth and calculating the mean PPD, we ensured a thorough characterization of periodontal status and treatment outcomes at the individual tooth level, thus facilitating a detailed analysis of the effectiveness of the interventions in managing periodontitis.

#### Microbiological assessment

Microbiological assessment was conducted at various time points: baseline, immediately after treatment, 2, 4, and 6-weeks post-treatment.

#### Sample collection and processing

The process of sample collection and processing included isolating and drying the specified area using a cotton swab (excluding the use of air syringe). A sterile paper point with the number 40 was then inserted into the base of the pocket until resistance was encountered, and it was left in place for 30 s. Care was taken during the removal of points to avoid salivary contamination. These points were meticulously placed in a securely wrapped sterile tube designed for transporting samples to a specialized laboratory [30].

Gingival sulcular fluid (GCF) samples were collected from all participants. Subsequent to collection, the GCF samples were deposited in a vial containing three milliliters of transport medium. Within 24 h of collection, these samples underwent processing. The samples were conveyed to the Medical, Microbiology, and Immunology department at the Faculty of Medicine, Mansoura University. The cones in the transport medium underwent a 30-min incubation at 37 °C to liquefy the jelly, following which they were promptly homogenized using tube agitators (Fisher Vortex Genie 2, USA).

#### 2- Bacterial identification

Bacterial identification involved identifying P.G and A.A culture. [31]

- A- Culture and identification of P.G: The samples were cultured on Petri dishes containing brucella blood agar. The total count of bacterial colonies was counted after incubation in an anaerobic environment at 37 °C for 7 days (Anaerobic Jar 2.5 L, Oxoid). Identification of P.G was based on the morphological and biochemical characteristics of the colonies. [31] P.G produced black colonies with no-hemolysis on blood agar. Gram staining was performed for the grown colonies; P.G is Gram-negative coccobacilli. It is indole positive, catalase negative, urease negative, and negative for the motility test [32].
- B- Culture and identification of AA:  The specimens were introduced onto Petri dishes containing Trypticase soy agar supplemented with horse serum, bacitracin, and vancomycin (referred to as TSBV-selective culture medium) as well as blood agar. Following a three-day incubation period at 37 °C in an environment with 5–10% carbon dioxide, the colonies underwent enumeration, and the presumed detection of A.A was based on the morphological and biochemical characteristics of the colonies. [31] A.A. produced white to grey smooth, convex and round colonies and β-hemolysis on blood agar. Gram staining shows Gram-negative coccobacilli. It is catalase positive, oxidase, urease, indole negative. It can ferment glucose, maltose, and mannite. It is unable to ferment lactose and sucrose which can differentiate it from A. Aphrophilus [33].

The colony-forming units (CFU) of P.G and A.A for each sample were enumerated on all blood agar plates. The average CFU/ml was recorded for each sample before and after the treatment regimen.

### Statistical analysis

Version 20.0 of the IBM SPSS software program (Armonk, NY: IBM Corp.) was used to conduct the analysis. Numbers and percentages were used to represent descriptive statistics for the qualitative data, and the Shapiro–Wilk test was used to determine if the distribution was normally distributed. The results obtained were deemed significant at the 5% level of evaluation.

## Results

The current study was conducted in 24 patients with stages I & II periodontitis according to the recent classification of periodontal diseases [34]. The following clinical indices were evaluated for each patient: PI, GI, PPD, and CAL. These indices were evaluated for all groups at baseline and further reevaluated again after six weeks following treatment. Furthermore, microbiological evaluation of the count of A. A and P.G in GCF was performed for all groups at baseline, immediately after treatment, 2, 4 and 6-weeks post treatment for all groups. We detected non statistically significant differences in the age and gender among the studied groups. Table 1

**Table 1:** Demographic data of all studied groups

Demographic data	Laser (*n* = 8)		Erythritol (*n* = 8)		Control (*n* = 8)		Test of significance	*p*- value
	**No**	**%**	**No**	**%**	**No**	**%**		
**Sex**								
Male	4	50.0	3	37.5	4	50.0	χ^2^= 0.446	^MC^*p* = 1.000
Female	4	50.0	5	62.5	4	50.0		
**Age (years)**								
Mean ± SD	40.12 ± 10.47		40.0 ± 11.93		35.50 ± 6.26		F = 0.573	*P* = 0.573

**SD:** Standard deviation

**χ**^**2**^**:** Chi square test

**MC:** Monte Carlo

**F**: F for One way ANOVA test

^*^: Statistically significant at *p* ≤ 0.05

Three groups in this study had their plaque indices assessed both before and after therapy. Prior to therapy, the plaque indices of each group were comparable. Plaque indices significantly decreased for all groups following treatment (*p* < 0.001), and there was no significant difference in post-treatment plaque indices between the three groups (*p* > 0.05).

Before therapy, there was no discernible difference between the three groups' gingival indices. Gingival indicators decreased significantly (*p* < 0.001) in all groups following treatment. The erythritol and control groups did not, however, differ significantly (*p* = 0.884). There were no significant differences between the three groups when the baseline probing depth was evaluated (*P* > 0.05).

All groups showed a significant decrease in PPD (*P* < 0.001) after treatment. Between the erythritol and control groups, there was no discernible change in PPD (*p* > 0.05). Following the intervention, CALs decreased significantly in every group (*P* < 0.05). Following the intervention, the CAL of the laser group was notably lower than that of the erythritol group (*P* = 0.004) and the control group (*P* = 0.028). Between the erythritol and control groups, there was no discernible change in CAL (*P* > 0.05) Table 2.

**Table 2:** Clinical indices of all studied groups at base line and 6 weeks after treatment

Indices	Laser Group *n* = 8	Erythritol Group *n* = 8	Control Group *n* = 8	F	*p*	Difference between groups
**Plaque index** **Before treatment** Mean ± SD				0.467	0.633	
	2.13 ± 0.13	2.13 ± 0.13	2.19 ± 0.18			
**After treatment** Mean ± SD				0.188	0.831	
	0.86 ± 0.24	0.86 ± 0.24	0.79 ± 0.27			
**t (p**_**0**_**)**	19.718^*^(< 0.001^*^)	19.718^*^(< 0.001^*^)	26.870^*^(< 0.001^*^)			
**Gingival index** **Before treatment** Mean ± SD				0.936	0.408	***P*1 = **0.010* ***P*2 = **0.004* ***P*3 = **0.884
	2.22 ± 0.21	2.28 ± 0.43	2.47 ± 0.45			
**After treatment** Mean ± SD				8.550^*^	0.002^*^	
	0.36 ± 0.24	0.86 ± 0.24	0.93 ± 0.35			
**t (p**_**0**_**)**	37.477^*^(< 0.001^*^)	7.325^*^(< 0.001^*^)	11.520^*^(< 0.001^*^)			
**Periodontal Probing depth** **Before treatment** Mean ± SD	2.94 ± 0.83	3.45 ± 0.87	3.34 ± 0.84	0.806	0.460	***P*1** = 0.530 ***P*2** = 0.004* *P***3** = 0.045*
**After treatment** Mean ± SD				7.217^*^	0.005^*^	
	0.86 ± 0.38	1.07 ± 0.37	1.58 ± 0.35			
**t (p**_**0**_**)**	10.155^*^(< 0.001^*^)	10.806^*^(< 0.001^*^)	10.043^*^(< 0.001^*^)			
**CAL** **Before treatment** Mean ± SD	2.52 ± 0.92	2.77 ± 1.04	2.36 ± 0.90	1.129	0.569	***P*1** = 0.517 ***P*2** = 0.004^*^ ***P*3** = 0.028^*^
**After treatment** Mean ± SD				8.918^*^	0.012^*^	
	0.83 ± 0.36	1.05 ± 0.57	1.68 ± 0.35			
**Z (p**_**0**_**)**	2.371^*^(0.018^*^)	2.366^*^ (0.018^*^)	2.366^*^(0.018^*^)			

*IQR* Inter quartile range, *SD* Standard deviation, Z Wilcoxon signed ranks test

**P:** *P* value for comparing between the three studied groups

**P**_**0**_**:** *P* value comparing before treatment and after treatment in each group

**P**_**1**_**:** *P* value compared between Laser group and Erythritol group after treatment

**P**_**2**_**:** *P* value comparing between Laser group and Control group after treatment

**P**_**3**_**:** *P* value comparing between Erythritol group and Control group after treatment

^*^: Statistically significant at *p* ≤ 0.05

**Fig. 1:**
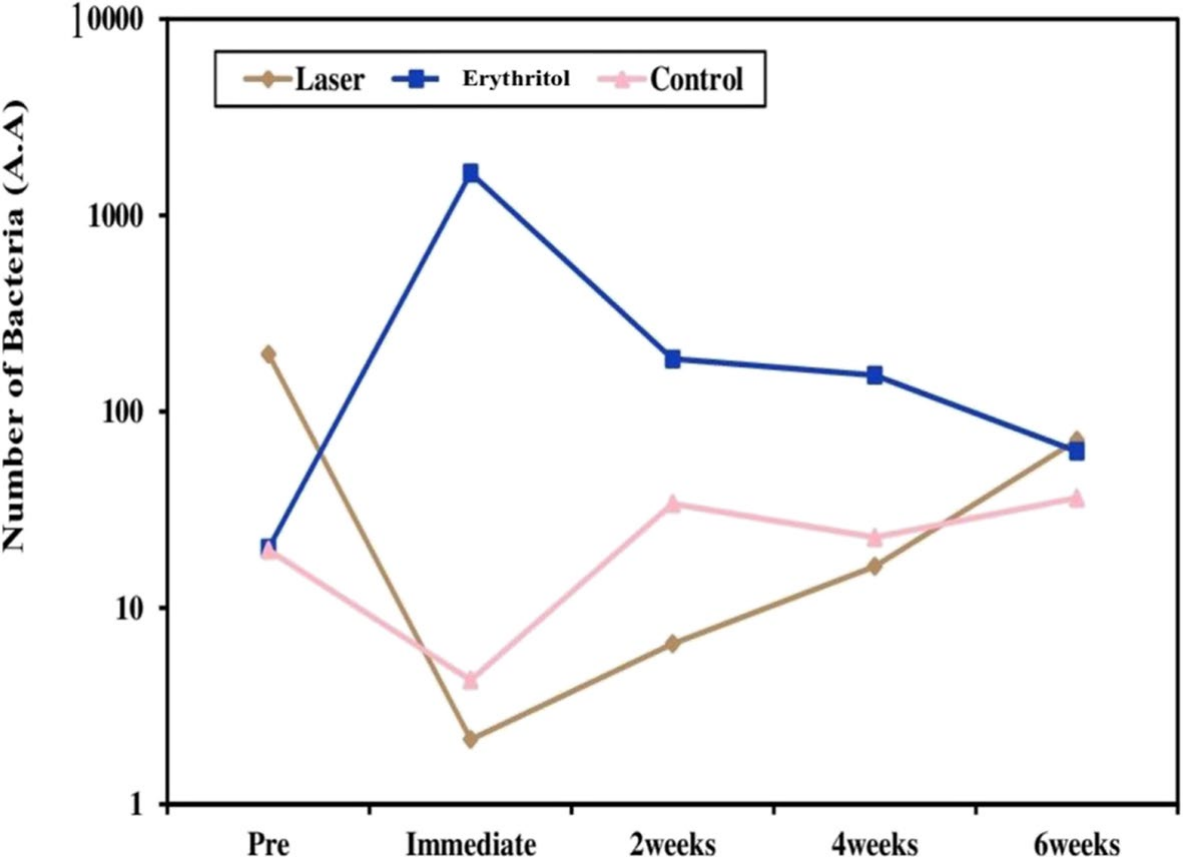
Microbiological comparison of the count of A.A between the three studied groups

At the pretreatment evaluation, there was no statistically significant difference in the count of A.A between the three examined groups (laser, erythritol, and control). We found that there was a significant difference in the count of A. A. across the three groups immediately following treatment (*P* = 0.001). In the laser group, the count of A.A. greatly dropped (*P* = 0.001), whereas in the erythritol group, it significantly rose (*P* = 0.014). We discovered a statistically significant difference between the laser and erythritol groups as well as erythritol and control groups (*P* < 0.05), however there was no statistically significant difference between laser and control group. There were no discernible variations in A.A. counts across the three groups at the 2-week post-treatment point. On the other hand, a substantial change was noted for the laser group between pretreatment and two weeks posttreatment.The microbial levels in all groups showed a trend of fluctuation over the next weeks after treatment, although there were no statistically significant differences (*P* > 0.05) Table 3 and Fig. 1.

**Table 3:** Comparison between the three studied groups according to microbiological count of A.A

Microbiology (A.A)	Laser group (*n* = 8)	Erythritol group (*n* = 8)	Control group (*n* = 8)	H	p	Difference between groups
**Pretreatment**						
Min. – Max	10 –1000	10–1000	0 – 100	3.967	0.07	
Median (IQR)	100(30.5 – 100)	0.0 (0.0 – 20.50)	0.0 (0.0 – 19.0)			
** Immediate after treatment**						p_5_ = 0.002^* ^p_6_ = 0.816
Min. – Max	0.0 – 12	10 – 10,000	0.0 – 30	14.442^*^	0.001^*^	p_7_ = 0.001
Median (IQR)	0.0 (0.0 – 1.50)	100(100–600)	0.0 (0.0 – 0.0)			
**2 weeks post treatment**						
Min. – Max	0.0 – 30	0.0 – 1000	0.0 – 100	2.780	0.249	
Median (IQR)	4 (0.0 – 6.0)	80(9.50 – 100.0)	17 (0.0 – 60.50)			
**4 weeks post treatment**						
Min. – Max	0.0 – 100	0.0 – 1000	0.0 – 100	0.124	0.940	
Median (IQR)	3 (1.0 – 4.50)	3 (0.0 – 35.0)	9(0.0 – 25.50)			
**6 weeks post treatment**						
Min. – Max	0.0 – 200	0.0 – 300	0.0 – 100	0.008	0.996	
Median (IQR)	0.0 (0.0 – 150)	15(0.0 – 62.50)	20 (2.0 – 65)			
** Fr**	14.783^*^	10.299^*^	4.807			
** p**_**0**_	0.005^*^	0.036^*^	0.308			
** p**_**1**_	0.001^*^	0.014^*^				
** p**_**2**_	0.009^*^	0.447				
** p**_**3**_	0.052	0.735				
** p**_**4**_	0.063	0.612				

H: H for Kruskal Wallis test, Fr: Friedman test

**p:** p value for comparing between the three studied groups

**p**_**0**_**:** p value for comparing between the different studied periods in each group

**p**_**1**_**:** p value for comparing between Pretreatment and Immediate after treatment

**p**_**2**_**:** p value for comparing between Pretreatment and 2 weeks after treatment

**p**_**3**_**:** p value for comparing between Pretreatment and 4 weeks after treatment

**p**_**4**_**:** p value for comparing between Pretreatment and 6 weeks after treatment

**p**_**5**_**:** p value for comparing between Laser and Erythritol groups

**p**_**6**_**:** p value for comparing between Laser and Control groups

**p**_**7**_**:** p value for comparing Erythritol and Control groups

^*^: Statistically significant at p ≤ 0.05

**Fig. 2:**
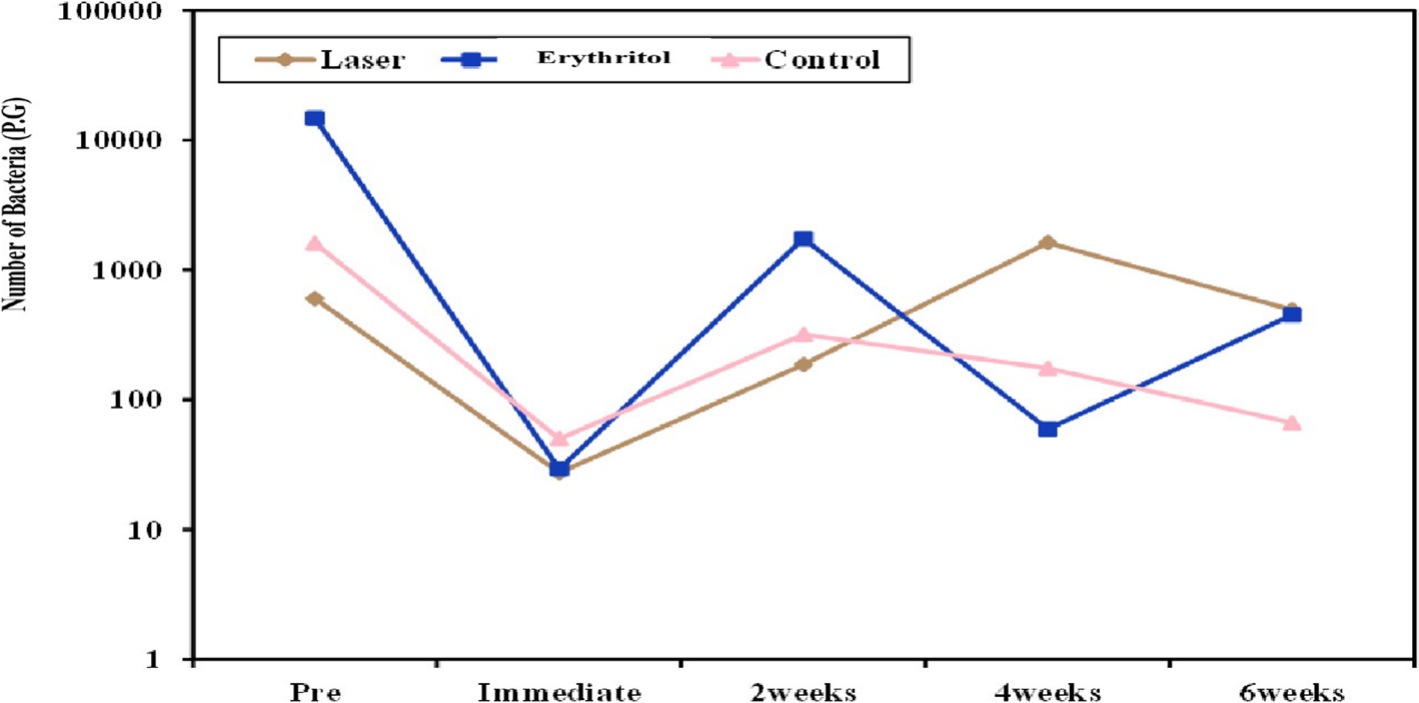
Comparison between the three studied groups according to microbiology (P.G)

The study compared the microbiological count of P.G. in three groups: laser, erythritol, and control, at various time intervals. Microbial levels varied a lot during the pretreatment stage; however, the data did not show any discernible differences between the three groups. Microbial levels decreased immediately after treatment in all groups. There were notable variations for the Laser group between pretreatment and the first posttreatment period (*P* = 0.028). Between pretreatment and the first posttreatment period, there was a significant difference for the Erythritol group (*P* = 0.001). Regarding the other comparisons, there were no noteworthy variations found. There were no discernible changes in the control group between treatment intervals. (*P* > 0.05). Table 4 and Fig. 2.

**Table 4:** Comparison between the three studied groups according to microbiology (P.G)

Microbiology (P.G)	Laser group (*n* = 8)	Erythritol group (*n* = 8)	Control group (*n* = 8)	H	p
**Pretreatment**					
Min. – Max	50 – 1000	25 – 100,000	0 – 10,000	1.824	0.402
Median (IQR)	1000 (80 – 1000)	1000(100 – 1000)	100 (20 – 550)		
**Immediate post treatment**					
Min. – Max	0 – 100	0 – 100	0.0 – 100	1.358	0.507
Median (IQR)	0.0 (0.0 – 46)	0.0 (0.0 – 52.50)	50 (0.50 – 100)		
**2 weeks posttreatment**					
Min. – Max	0.0 – 1000	30.0 – 10,000	0.0 – 1000	1.697	0.428
Median (IQR)	80 (13 – 100)	100 (40 – 1000)	100(15.5 – 550)		
**4 weeks posttreatment**					
Min. – Max	17 – 10,000	16 – 100	0 – 1000	2.789	0.248
Median (IQR)	100 (70.0 – 550.0)	40 (30 – 100)	40 (16.50 – 75.0)		
**6 weeks posttreatment**					
Min. – Max	50 – 1000	5.0 – 1000	19 – 150	4.357	0.113
Median (IQR)	200(100–1000)	100 (29.5 – 1000)	50 (22.5 – 100)		
** Fr**	10.646	13.221^*^	2.168		
** p**_**0**_	0.031^*^	0.010^*^	0.705		
** p**_**1**_	0.028^*^	0.001^*^	–		
** p**_**2**_	0.205	0.612	–		
** p**_**3**_	0.866	0.076	–		
** p**_**4**_	0.735	0.205	–		

IQR: Inter quartile range

**Fr**: Friedman test

**p:** *p* value for comparing between the three studied groups

**p**_**0**_**:** *p* value for comparing between the different studied periods in each group

**p**_**1**_**:** *p* value for comparing between **Pretreatment** and **Immediate after treatment**

**p**_**2**_**:** *p* value for comparing between **Pretreatment** and **2 weeks after treatment**

**p**_**3**_**:** *p* value for comparing between **Pretreatment** and **4 weeks after treatment**

**p**_**4**_**:** *p* value for comparing between **Pretreatment** and **6 weeks after treatment**

^*^: Statistically significant at *p* ≤ 0.05

## Discussion

Periodontitis, a multifaceted inflammatory condition, results in the deterioration of supporting periodontal tissues. The primary objective of periodontal therapy is to eradicate bacterial plaque and the contributing factors to its deposition [35, 36]. The aim of the current study is to assess the impact of diode laser and subgingival air polishing with erythritol in the treatment of stage I and stage II periodontitis. A total of twenty-four individuals were recruited and evenly assigned to three groups. The participants, irrespective of gender, were randomly selected, with no sex predilection, and aged 30 years or older. In our investigation we focused on P.G and A.A to assess the effect of diode laser and subgingival air polishing on healing of periodontal disease. Previous research indicates that P.G. is more frequently present and at higher levels in sites exhibiting signs of active disease [37]. In addition, A.A which was considered the causative agent of aggressive periodontitis [38].

The choice of these bacteria was based on a strong association between their high levels in GCF, and their impact on clinical measures of periodontitis [39, 40]. While SRP remains the conventional therapeutic modality of periodontal disease [41], lasers play a pivotal role in periodontal therapy. They contribute to the reduction of bacteria [42–45], and enhancement of periodontal regeneration in humans, all without causing harm to the surrounding bone and pulp tissues. [46–48]. In our study, a diode laser was chosen as it is one of the most popular laser technologies, known for its low cost, portability, and ease of use [49].

The laser utilized in this study featured an infrared wavelength of 940 nm, offering superior penetration depth compared to lasers operating within the visible spectrum [50]. This wavelength falls within the range of 800–980 nm, a spectrum well-absorbed by pigmented tissues. This characteristic facilitates the selective targeting of darkened, inflamed tissues, and pigmented bacteria. It is noteworthy that various trials have unequivocally substantiated the bactericidal efficacy of the diode laser [12, 43, 51–53]. The assessment of the PI aimed to evaluate the oral hygiene status of the patients and also to evaluate the effect of diode laser on the plaque revealing a notable decrease posttreatment (*P*0 < 0.001). However, there was no discernible advantage of laser over the control group, attributed to the practice of re-motivating the patient at each recall interval, a measure that was duly implemented.

The evaluation of the GI aimed to clinically assess the gingival condition. Our study unveiled a significant reduction in gingival inflammation in the laser-treated group compared to erythritol and control groups respectively (*P*1 = 0.010, P2 = 0.004), with noteworthy differences observed at follow-up (*P*0 < 0.001). The enhancement of the G.I. in the laser group, as opposed to the erythritol and control groups, can be attributed to the ability of diode laser to reduce inflammation [54].

We measured the effect of the different treatment modalities utilized in the current study on the periodontal pocket depth which plays a pivotal factor influencing the long-term stability of results. In addition, PPD served as a primary outcome in our measurements. Additionally, CAL was closely examined. The use of a diode laser as an adjunct therapy revealed significant statistical differences in these parameters: PPD (*P*0 < 0.001) and CAL (*P*0 = 0.018) after 6 weeks follow-up. Our study demonstrated a noteworthy reduction in PPD and CAL in the laser-treated group compared to the control group (*P*2 = 0.004). This reduction can be explained by the diode laser's potential "guided tissue regeneration-like" effect, impeding epithelial migration and potentially achieving more thorough pocket epithelial removal compared to conventional mechanical methods, as demonstrated in vitro [55]. Additionally, the diode laser neutralizes bacterial toxins within the root cementum, so unquestionably promoting periodontal health. [30]

The findings of this study are consistent with those of Dakhil & Mahmood, 2020 [56], Lobo & Pol, 2015 [12] who used 940 nm and 1.5 Watt. Crispino et al., 2015 who used 940 nm and 3 Watt [57], Tabari et al., 2021 who utilized a diode laser with 940 nm, 1 Watt [58] and ODOR et al., 2018 who employed a diode 940 nm laser,1.1 Watt [59]. All of these studies assessed the application of diode laser with SRP compared to SRP alone. They reported a significant reduction in all clinical indices (PI, GI, PPD, CAL), with the laser group demonstrating greater improvement across all indices in comparison to the SRP group. This pattern included a more effective restoration of gingival health.

On the contrary, the study conducted by Micheliet et al. 2011, which assessed the application of diode laser with SRP compared to SRP alone, reported no significant reduction in all clinical indices. [31] Slot et al., 2014 which evaluated the application of diode laser with SRP compared to SRP alone, reported no significant reduction in PPD and CAL [60].

In recent times, a novel air-polishing device utilizing erythritol powder has been introduced in supportive periodontal therapy. It can be successfully used in a safe non-surgical periodontal therapy for biofilm control [18]. The analysis of secondary outcome variables, PI and GI revealed a notable decrease post-treatment (P0 < 0.001). However, there was no significant statistical difference between the erythritol and control groups. This may be attributed to the re-motivation of the patient at each recall interval, a practice that was duly implemented.

The evaluation of primary outcome variables, PPD and CAL revealed a significant statistical difference at follow-up: PPD (*P*0 < 0.001) and CAL (*P*0 = 0.018). Simultaneously, the erythritol group recorded a better response than the control group at these parameters: PPD (*P*3 = 0.045) and CAL (*P*3 = 0.028). This superior response can be attributed to the efficacy of erythritol in gingival biofilm removal due to its small particle size and stable chemical properties compared to glycine [18, 61]. Additionally, erythritol exhibits effectiveness against certain periodontal bacteria, including P.G [62]. Significantly, the utilization of erythritol has demonstrated enhanced comfort and time efficiency [63]. The outcomes of this study align with those of Resnik et al. [15], Jentsch et al. [64], Cosgarea et al. [65], Hägi et al. [18], all of whom assessed the application of subgingival air-polishing with erythritol, and SRP compared to SRP alone. These studies reported a significant reduction in all clinical indices.

In contrast, the studies conducted by Onisor et al. [66], Mensi et al. [67], which assessed the application of subgingival air-polishing with erythritol, and SRP compared to SRP alone, reported no significant reduction in all clinical indices and no additional benefits to SRP.

Commensal bacteria play a crucial role in maintaining oral health by contributing to the overall balance and stability of the oral microbiome. These beneficial bacteria coexist harmoniously with the host, aiding in various functions such as digestion, immunity, and protection against pathogens. Thus, the presence of commensal bacteria is essential for the prevention of oral diseases, promoting overall oral health and well-being [68, 69].

Distinguishing between commensal bacteria and periodontal pathogens is paramount in understanding the intricate dynamics of oral health and disease [69]. In our study, we recognize the critical importance of targeting specific pathogenic species implicated in periodontal inflammation and tissue destruction. By focusing on these pathogens, such as A.A and P.G, we aim to address the cause of periodontal disease and develop targeted interventions to mitigate their harmful effects. By reducing the burden of periodontal pathogens while preserving beneficial commensal bacteria, our study contributes to promoting a healthier oral microbiome and improving overall periodontal health outcomes.

In the laser group, the microbiological analysis of A.A. and P.G. immediately after the application of the laser revealed significant statistical differences for both A.A. (*P*1 = 0.001) and P.G. (*P*1 = 0.028). And at 2 weeks Follow-up of A.A. (*P*2 = 0.009) In the case of A.A, the significant difference persisted for 2 weeks after treatment, more than P.G., which disappeared. The laser had a superior effect on A.A. than erythritol (*P*5 = 0.002) [30]. Laser radiation is absorbed by tissue chromophores, which include water, apatite minerals, and other pigments, in the target tissue. This is how lasers affect dental soft tissue and bacteria. One potential mechanism of laser action is photothermal ablation, in procedures using high-powered lasers, tissue is vaporized or coagulated as the laser is absorbed by a substantial tissue component. This leads to the breakdown of the bacterial cell wall, compromising bacterial integrity, causing an accumulation of denatured proteins, and ultimately resulting in cell lysis and microbial death [70].

These outcomes align with the findings of ODOR et al., who used a diode 940 nm laser, 1.1 W [59]. Additionally, Ciurescu et al. utilized the combined 2780 nm Er,Cr:YSGG and 940 nm In GaAsP diode laser, while Agarwal et al. employed a 940 nm diode laser [71, 72]. In their evaluation of diode laser application in the treatment of periodontitis compared to SRP alone, these studies reported a significant reduction in the count of A.A. and P.G.. In contrast to our study, the results of Alves VT et al. demonstrated that six months after baseline, both SRP and the combination of SRP with diode laser irradiation (808 ± 5 nm, 1.5 Watt) yielded similar benefits in the studied microbiological aspect, specifically a decrease in the count of A.A. and P.G. No statistical difference was observed between the groups. This observation is linked to the variation in energy measured at the tip of the fiber optics, emphasizing the need for compensation with the aid of a power meter [21].

In the erythritol group, no statistically significant differences between groups were observed at baseline for A.A. and P.G. However, following the application of erythritol, a significant statistical difference emerged for A.A. (*P*1 = 0.014), indicating an immediate increase in bacterial count. The rise in A.A. bacteria count immediately after the application of erythritol may be attributed to the disruption of microbial biofilm. Biofilms, which are communities of bacteria adhering to surfaces and surrounded by an extracellular polymeric substance matrix, are known for their resistance to host immune responses and antimicrobial medications. Research has shown that erythritol disrupts biofilms produced by various bacteria, including A.A. This disruption causes individual bacteria to detach from the biofilm matrix, potentially leading to a temporary increase in their numbers in the surrounding area [73]. However, this disruption ultimately weakens the overall bacterial community, rendering it more susceptible to further antimicrobial actions, including those of erythritol itself or other agents.

In the case of P.G., an immediate and significant statistical difference was observed following the application of erythritol (*P*1 = 0.001). This reduction in bacterial count underscores the pronounced inhibitory effect of erythritol on both P.G heterotypic biofilm development and bacterial growth. Erythritol induced notable alterations in the microstructures of biofilms formed by both species, leading to a greater reduction in biovolumes compared to other sugar alcohols. Furthermore, the surface-associated Rgp activity (Relaxin-like Gonad-Stimulating Peptides) of P. gingivalis was also suppressed. These findings collectively suggest that erythritol exerts multiple suppressive effects on the P.G heterotypic community [19]. These outcomes align with the findings of Park et al., Jentsch et al., and ZHANG &YAO, who, in their evaluation of erythritol application in the treatment of periodontitis compared to SRP alone, reported a significant reduction in the count of A.A. and P.G [64, 74, 75].

In contrast, the study conducted by Resnik et al., which assessed the application of subgingival air-polishing with erythritol, and SRP compared to SRP alone, reported no significant reduction in bacterial count(A.A. and P.G.) and no additional benefits to conventional periodontal treatment [15]. Moreover, no significant difference was observed between laser and erythritol in terms of PPD and CAL, highlighting the value of erythritol as a treatment for periodontitis.

## Conclusions

The outcomes of the current study revealed that the application of diode laser and erythritol air polishing have a beneficial effect in the treatment of periodontitis compared to SRP alone.

## Limitations

It is crucial to acknowledge certain limitations of the study. Firstly, the sample size was relatively small. This may impact the generalizability of the findings to a larger population. Additionally, the study focused on patients with stages I & II periodontitis, limiting the applicability of the results to more advanced cases. Furthermore, the follow-up period of six weeks may be considered relatively short, and a longer-term assessment could provide a more comprehensive understanding of treatment outcomes. Future research with larger and more diverse populations, including longer-term follow-ups, would contribute to a more robust understanding of the outcomes of diode laser and erythritol air polishing in periodontal treatment.

## Data Availability

The data that support the findings of this study are available from the corresponding author upon reasonable request.
